# Supplementary material for: OptoCENTAL: a standardized, bench-testing platform based on phantoms for validating optical systems aimed at clinical monitoring of the placenta
Source: J Biomed Opt. 2026 Sep 26;31(9):097004. doi: 10.1117/1.JBO.31.9.097004 (PMC13621447; doi:10.1117/1.JBO.31.9.097004)
Supplement: Supplementary file 1 [file JBO_031_097004_SD001.pdf]

OptoCENTAL: a standardised, bench-testing platform based on phantoms for validating optical systems aimed at clinical monitoring of the placenta

Luca Giannoni,[a*] Uzair Hakim,[a] Fréderic Lange,[a] Musa Talati,[a] Darshana Gopal,[a] Angelos Artemiou,[a] Niccole Ranaei-Zamani,[b] Subhabrata Mitra, [b] Ilias Tachtsidis[a]
[a] University College London, Department of Medical Physics and Biomedical Engineering, London, UK
[b] University College London, Neonatology, EGA Institute for Women's Health, London, UK

Keywords: biomedical optics, NIRS, cytochrome-c-oxidase, placental monitoring, optical phantoms.

***Luca Giannoni**, E-mail: l.giannoni@ucl.ac.uk

Supplementary Materials

Table S.1 reports the numerical results of the sensitivity estimated in PROTOCOL 0 for MAESTROS II, while Table S.2 and S.3 reports them for the FetalSenseM V.15 and V2, respectively

Table S.1 Numerical results for example sensitivity profiles of MAESTROS II from PROTOCOL 0.

Sensitivity (%) to specific layers - Detector 1					
**Bin start (ns)**	**Bin end (ns)**	**Skin**	**Adipose**	**Muscle**	**Placenta**
0.23	0.41	5.00	5.38	14.33	75.30
0.43	0.61	4.80	5.20	13.93	76.06
0.63	0.81	4.64	5.04	13.56	76.76
0.83	1.01	4.49	4.89	13.20	77.43
1.03	1.21	4.37	4.74	12.85	78.04
1.23	1.41	4.21	4.61	12.53	78.65
1.43	1.61	4.08	4.48	12.23	79.21
1.63	1.81	3.96	4.36	11.94	79.75
1.83	2.01	3.85	4.24	11.66	80.24
2.03	2.21	3.76	4.14	11.39	80.71
2.23	2.41	3.64	4.04	11.14	81.18
2.43	2.61	3.54	3.94	10.90	81.62
2.63	2.81	3.46	3.85	10.66	82.03

Sensitivity (%) to specific layers - Detector 2					
**Bin start (ns)**	**Bin end (ns)**	**Skin**	**Adipose**	**Muscle**	**Placenta**
0.39	0.57	4.84	5.22	13.99	75.95
0.59	0.77	4.66	5.06	13.61	76.67
0.79	0.97	4.52	4.90	13.25	77.33
0.99	1.17	4.37	4.76	12.91	77.97
1.19	1.37	4.21	4.62	12.58	78.59
1.39	1.57	4.09	4.49	12.27	79.14
1.59	1.77	3.95	4.37	11.98	79.70
1.79	1.97	3.85	4.26	11.70	80.19
1.99	2.17	3.75	4.15	11.43	80.67
2.19	2.37	3.65	4.05	11.17	81.13
2.39	2.57	3.58	3.95	10.93	81.54
2.59	2.77	3.46	3.86	10.70	81.98
2.79	2.97	3.39	3.77	10.47	82.37
2.99	3.17	3.31	3.68	10.26	82.75
3.19	3.37	3.24	3.59	10.05	83.12
3.39	3.57	3.17	3.52	9.85	83.45

Sensitivity (%) to specific layers - Detector 3					
**Bin start (ns)**	**Bin end (ns)**	**Skin**	**Adipose**	**Muscle**	**Placenta**
0.57	0.75	4.66	5.05	13.63	76.66
0.77	0.95	4.49	4.90	13.27	77.35
0.97	1.15	4.38	4.76	12.92	77.94
1.17	1.35	4.24	4.62	12.60	78.54
1.37	1.55	4.11	4.49	12.29	79.11
1.57	1.75	3.99	4.37	11.99	79.64
1.77	1.95	3.89	4.26	11.71	80.15
1.97	2.15	3.77	4.15	11.44	80.63
2.17	2.35	3.66	4.05	11.19	81.11
2.37	2.55	3.56	3.95	10.94	81.55
2.57	2.75	3.48	3.86	10.71	81.96
2.77	2.95	3.36	3.76	10.49	82.39

2.97	3.15	3.30	3.68	10.27	82.75
3.17	3.35	3.24	3.60	10.06	83.09
3.37	3.55	3.17	3.52	9.87	83.44
3.57	3.75	3.07	3.45	9.68	83.80
3.77	3.95	3.00	3.37	9.49	84.13
**Detector 4**					
**Bin start (ns)**	**Bin end (ns)**	**Skin**	**Adipose**	**Muscle**	**Placenta**
0.59	0.77	4.62	5.00	13.56	76.82
0.79	0.97	4.45	4.86	13.20	77.49
0.99	1.17	4.34	4.72	12.86	78.08
1.19	1.37	4.19	4.59	12.54	78.68
1.39	1.57	4.06	4.46	12.23	79.25
1.59	1.77	3.94	4.34	11.94	79.78
1.79	1.97	3.84	4.23	11.66	80.27
1.99	2.17	3.74	4.12	11.40	80.74
2.19	2.37	3.63	4.02	11.15	81.20
2.39	2.57	3.54	3.92	10.90	81.64
2.59	2.77	3.45	3.83	10.67	82.05
2.79	2.97	3.36	3.74	10.45	82.45
2.99	3.17	3.29	3.66	10.24	82.81
3.19	3.37	3.22	3.58	10.03	83.16
3.39	3.57	3.14	3.51	9.84	83.51
3.59	3.77	3.07	3.44	9.65	83.84
3.79	3.97	2.99	3.37	9.47	84.17
3.99	4.17	2.94	3.29	9.29	84.48
4.19	4.37	2.88	3.23	9.12	84.77

Table S.2 Numerical results for example sensitivity profiles of the FetalSenseM V1.5 from PROTOCOL 0.

**Sensitivity (%) to specific layers - FetalSenseM V1.5**				
**Detector**	**Skin**	**Adipose**	**Muscle**	**Placenta**
3cm	8.70	24.90	49.61	16.79
5cm	5.28	16.99	45.95	31.78

Table S.3 Numerical results for example sensitivity profiles of the FetalSenseM V2from PROTOCOL 0.

Sensitivity (%) to specific layers - FetalSenseM V2				
**Detector**	**Skin**	**Adipose**	**Muscle**	**Placenta**
3cm	4.04	7.54	9.54	54.93
4cm	3.04	6.08	8.27	62.45
5cm	2.43	4.73	6.80	69.43

The following sections of the Supplementary Materials report the theory and algorithms used for the SRS and DS analysis performed on all the datasets reported in PROTOCOL 0 and PROTOCOL 2 of the OptoCENTAL testing platform.

S.1 Spatially Resolved Spectroscopy (SRS)

The SRS algorithm is able to compute absolute tissue haemoglobin saturation (and therefore oxygen saturation) by making use of multi-distance source-detector configurations. Such method was used with the FetalSenseM V1.5 and V2 devices. Both devices utilise multi-distance source detector separations (Table 8), and for both systems the mean distance (ρ) was taken to be 4 cm.

S. 1.1 Theory

The propagation of photons in a highly scattering medium (i.e. tissue) can be described by the diffusion approximation. In the case of CW NIRS, the intensity of the light can be expressed as the integral of reflected light, *R*, at a given source-detector separation and time:

$$I(\rho) = \int_0^\infty R(\rho, t)dt \qquad (S.1)$$

And so, the attenuation at a source-detector separation, ρ, can be expressed as:

$$A(\rho) = -log_{10} \int_0^\infty R(\rho, t)dt \qquad (S.2)$$

Given that the reflected light, *R*, can be described by the diffusion approximation, if Eq. S.2 is

differentiated with respect to ρ, Eq. S.3 is obtained.

$$\frac{\delta A}{\delta \rho} = \frac{1}{\ln(10)} \cdot \left(\sqrt{3\mu_a \cdot \mu'_s} + \frac{2}{\rho}\right) \qquad (S.3)$$

The absorption coefficient can be obtained by re-arranging Eq. S.3 to obtain μ_a as shown in Eq. S.4. Here, the scattering coefficient, μ_s is equivalent to $k \cdot (1 - h \cdot \lambda)$, where h is a measured quantity and is typically 6.3x10^{-4} mm^{-1}/nm for all tissue types[54].

$$k \cdot \mu_a(\lambda) = \frac{1}{3(1 - h \cdot \lambda)} \cdot (ln10 \cdot \frac{\delta A(\lambda)}{\delta \rho} - \frac{2}{\rho})^2 \qquad (S.4)$$

Once μ_a is obtained for the relevant number of wavelengths, then Modified-Beer Lambert's Law (MBLL) can be used to compute the concentrations. As can be seen in Eq. S.4, the parameter $\frac{\delta A(\lambda)}{\delta \rho}$ is required to compute kμ_a. $\frac{\delta A(\lambda)}{\delta \rho}$ is defined as the change in attenuation in relation to the source-detector separation, i.e. the gradient of how attenuation changes with changing source-detector separation. In theory if the gradient is high then the indication is that the recorded attenuation is sensitive to the changes in absorption from the deeper layers. Once the gradient is calculated and μ_a is calculated, Modified-Beer Lambert's Law (MBLL) can be used to calculate the concentrations of HbO_2 and HHb, and thus StO_2.

S.2 Dual Slope (DS)

The Dual Slope (DS) method is the most recently developed method of the ones discussed here[40]. Similar to the SRS the DS method also exploits the use of the gradient of datatype (i.e. attenuation) versus source-detector separation. The DS method is characterized by the implementation of multiple sources in an optode configuration. The use of data from two sources offers several advantages: (1) insensitivity to instrumental effects related to temporal variations in source emission and detector sensitivity properties. (2) Insensitivity to changes in optical coupling between optical

probe and tissue. (3) Reduced sensitivity to localized as well as uniform superficial tissue inhomogeneities, resulting in a greater relative sensitivity to deeper tissue. (4) Localised sensitivity to a deep tissue volume, which does not feature the typical banana shape of NIRS regions of sensitivity. The DS method was used with both the FetalSenseM V1.5 and V2 devices.

S. 2.1 Theory

In general, the theory behind the DS method follows the same logic as the SRS. The idea behind the DS method is to obtain two *matched* slopes, where each source and detector pairing is switched for each slope. In other words, a given source/detector that generates data at a short distance for one slope, simultaneously generates data at a long distance for the other slope. The detectors should be placed such that their distances from the two sources are symmetric with respect to the average source-detector distance. Sassaroli *et al* [40]. presents several equations serving the purpose to compute the slope for two sources and multiple detectors. Importantly, the proposed equations differ from the conventional slope calculation.

To compute the slope conventionally (i.e. for the SRS), one of two formulae can be used, given in Eq. S.5 where r_2 and r_1 are the source-detector distances of interest, and $DC(r_{1/2})$ is the intensity recorded at detector r_1 and r_2.

$$\text{Slope} = \frac{\ln\big(DC(r_2)\big) - \ln\big(DC(r_1)\big)}{r_2 - r_1} \qquad (S.5)$$

Eq. S.6 uses the standard form of a line to compute a linear fitting to the points defined by $ln(DC)$ at given source-detector separations, where the slope is defined by m. Either one of these methods can be used to compute the slope, however each have different capabilities.

$$ln(DC) = mr + c \qquad (S.6)$$

The key difference between these two methods is that equation S.5 is only able to compute the

slope for two detector spacings. In contrast Eq. S.6 can compute it for as many *r* values as desired. Firstly, as stated, Eq. S.5 relies on two detectors at a time and ignores the broader detector configuration. It does not utilize the symmetry of the system, and so effects from superficial artifacts persist. Additionally, it is highly sensitive to noise contained within the two chosen points. Secondly, Eq. S.6 assumes that all detectors contribute equally to the slope, ignoring variance or symmetry in the optical configuration. This means it treats all points as equally reliable, even if some measurements are noisier or further from the mean source-detector separation.

To overcome these, Sassaroli *et al*[40] provide an equation to compute the slope, Eq. S.7, *DC* here refers to the intensity measured at each detector:

$$SSl = \frac{\sum_{i=1}^{N}(r_i - \langle r \rangle)\ln\left(r_i^2 DC_1(r_i)\right)}{N \cdot \mathrm{var}(r)} \quad (S.7)$$

Eq. S.7 improves upon Eq. S.6 by introducing statistical weighting. Instead of assuming all detectors contribute equally to the slope, it weights the contribution of each detector based on its deviation from the mean source-detector separation ($\langle r \rangle$) and normalizes the slope using the variance of separations ($\mathrm{var}(r)$). This makes the calculation more robust to noise and uneven detector spacing. Furthermore, it is clear that Eq. S.7 weights the contribution of detectors further away from the source more than those nearer, thus aiding in increasing the depth sensitivity of the method.

For the two FetalSenseM devices, the mean source detector separation was 4 cm. The variance for the FetalSenseM V1.5 was 0.5 and for the V2 was 1.

Eq. S.7 is used for each source to obtain a slope for each source to the same detectors. Once a slope value is obtained for each source, these are averaged together to obtain Eq. S.8:

$$DSl = \frac{SSl_1 + SSl_2}{2} \quad (S.8)$$

Where $SSl_{1/2}$ refers to the single slope from source 1 and source 2. Importantly, the authors state

that for source 1/2 the role of the detectors must be switched. This means that a detector generating data at a short/long distance for one source, must generate data at a long/short distance for the other source.

Once the slopes are calculated, the method from Blaney *et al*.[55] can be used to obtain values for HbO_2 and HHb, which are then used to calculate StO_2. To calculate the absorption coefficient using a single slope (*SSl*), Eq. S.9 is used:

$$\Delta\mu_a = -\frac{SSl}{DSF} \qquad (S.9)$$

Which can be expanded to the dual-slope case:

$$\Delta\mu_a = -\frac{SSl_1 + SSl_2}{2 \times DSF} \qquad (S.10)$$

In both Eq. S.9 and S,10, *DSF* refers to the Differential Slope Factor, a parameter analogous to the DPF (differential path length factor) that is defined by Eq. S.11:

$$DSF = \frac{\sum_{i=1}^{n}(r_i - \langle r\rangle)\langle L_i\rangle\left(r_s, r_{d,i}\right)}{\sum_{i=1}^{n}(r_i - \langle r\rangle)^2} \qquad (S.11)$$

The *DSF* is a weighted sum of the mean pathlengths for each source-detector pair, where the weights are the deviations of a given source-detector separation (r_i) from the mean separation, representing how the mean pathlength ($\langle L_i\rangle\left(r_s, r_{d,i}\right)$) varies with source-detector separation and is normalized by the sum of squared deviations from the mean, to account for variability in source-detector separations. The mean pathlength here was acquired from previously conducted simulations. The *DSF* was taken to be 6.9 for both FetalSenseM devices.

Once a value for μ_a is computed, Modified-Beer Lambert's Law (MBLL) can be used to determine HbO_2 and HHb, and hence StO_2.

S.3 MRI-based heterogenous simulations

This simulation used a segmented MRI scan of a patient's abdomen for a patient at 30 weeks gestation with an anterior placenta. The MRI image was initially segmented by the UCLH clinical team using ITK Snap. From the segmented images the 3D mesh was generated using iso2mesh[56]. This outputs a structurally accurate 3D model to run simulations with. The pipeline to generate the 3D mesh is shown in Fig. S.1a. This mesh provides four tissues: (1) Adipose, (2) Muscle, (3) Placenta and (4) Other tissues (tissues below the placenta). Their optical properties are reported in Table S.4.

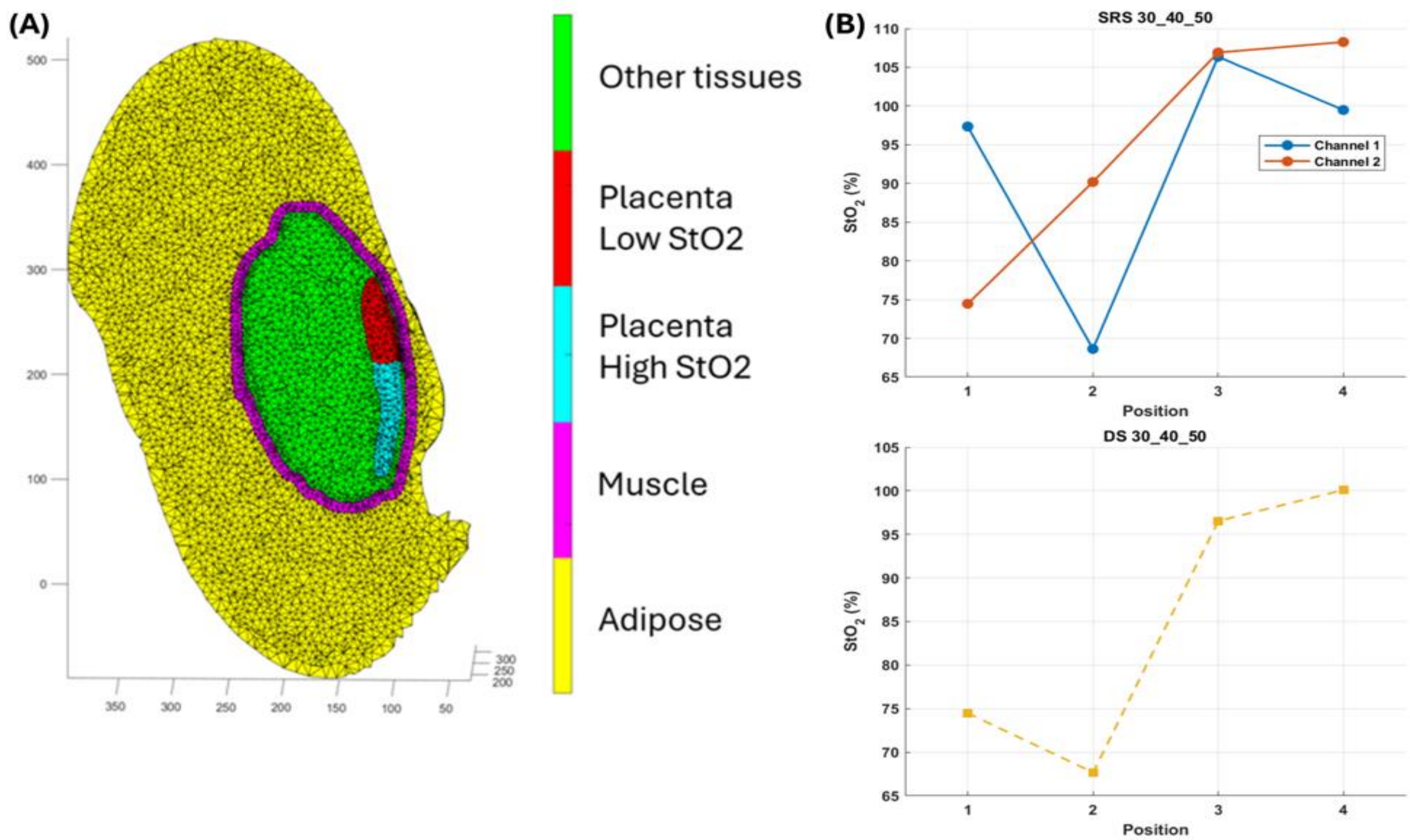

Fig. S.1 (a) 3D meshed geometry of heterogenous, anterior placenta and abdominal overlayers from an MRI scan of a patient taken at UCLH; (b) Oxygenation saturation determined by both the SRS (top) and DS (bottom) methods simulating the FetalSenseM V2 device. At position 1 the device was fully in the low StO_2section. At positions 2 and 3 the device was positioned such that the source and the detectors were in both low and high areas of StO_2, whilst position 4 was completely in the high StO_2 area.

The mesh-based simulations incorporated two discrete regions of oxygenation in the placenta, at StO_2 of 25% and 100%. The device was scanned downwards in steps of 3 cm, covering four positions on the abdomen, for the case of the FetalSenseM V2. The purpose of these simulations was to assess the ability of the device to detect variation in oxygenation as it is positioned at different points on the abdomen, starting in the area of low StO_2 and moving towards the high StO_2. Results for the example shown in Fig S.1a are reported in Fig. 1b, for both SRS and DS methods.

Table S.4 Optical properties of the tissue layers used in the MRI-based heterogenous model.

Tissue type	g	n	μ_a (mm^{-1})	μ_s (mm^{-1})
Adipose	0.9	1.37	0.002	13.6
Muscle	0.9	1.37	0.012	8.571
Placenta (Low)	0.9	1.4	0.0133	8.751
Placenta (High)	0.9	1.4	0.0109	8.751
Other Tissue	1	1	0	0

References (from main reference list)

40. A. Sassaroli, G. Blaney, and S. Fantini, “Dual-slope method for enhanced depth sensitivity in diffuse optical spectroscopy,” *J. Opt. Soc. Am. A Opt. Image Sci. Vis.* **36**(10), 1743 (2019) [doi:10.1364/JOSAA.36.001743].

54. S. Suzuki et al., “Tissue oxygenation monitor using NIR spatially resolved spectroscopy,” *Biomedical optics* **3597**, 582–592 (1999) [doi:10.1117/12.356862].

55. G. Blaney et al., “Phase dual-slopes in frequency-domain near-infrared spectroscopy for enhanced sensitivity to brain tissue: First applications to human subjects,” *J. Biophotonics* **13**(1) (2020) [doi:10.1002/JBIO.201960018].

56. A. P. Tran, S. Yan, and Q. Fang, "Improving model-based functional near-infrared spectroscopy analysis using mesh-based anatomical and light-transport models," Neurophotonics **7**(1), 015008 (2020 [doi: 10.1117/1.NPh.7.1.015008])
